# Evaluation of low cryptococcal antigen titer as determined by the lateral flow assay in serum and cerebrospinal fluid among HIV-negative patients: a retrospective diagnostic accuracy study

**DOI:** 10.1186/s43008-020-00028-w

**Published:** 2020-03-10

**Authors:** Xuan Wang, Jia-Hui Cheng, Ling-Hong Zhou, Jun-Hao Zhu, Rui-Ying Wang, Hua-Zhen Zhao, Ying-Kui Jiang, Li-Ping Huang, Ching-Wan Yip, Chun-Xing Que, Min Zhu, Li-Ping Zhu

**Affiliations:** 1 Department of Infectious Diseases, Huashan Hospital, Shanghai Medical College, Fudan University, 12 Central Urumqi Road, Shanghai, 200040 China; 2 Division of Mycology, Huashan Hospital, Shanghai Medical College, Fudan University, 12 Central Urumqi Road, Shanghai, 200040 China

**Keywords:** Cryptococcosis, Meningitis, Low cryptococcal antigen titer, Lateral flow assay, Diagnostic accuracy

## Abstract

Cryptococcosis is one of the most common opportunistic infections in both immunocompetent and immunocompromised hosts. Although the cryptococcal antigen (CrAg) lateral flow assay (LFA) has been widely used in clinical settings due to its high sensitivity and specificity, the diagnostic value of a low CrAg LFA titers remains unclear. In this study, we performed a retrospective analysis of 149 HIV-negative patients with low CrAg LFA titers (≤1:10) in a Chinese tertiary hospital from January 2013 to December 2017, to evaluate the diagnostic value of low CrAg LFA titers in serum and cerebrospinal fluid (CSF) at different thresholds. Sensitivity and specificity of low CrAg LFA titers in patients with definitive diagnoses of cryptococcosis were 39.6% (95% CI, 29.7–50.1%) and 100% (95% CI, 69.2–100%), respectively, at a threshold of 1:10 in serum. A sensitivity of 72.9% (95% CI, 62.9–81.5%) and a decreased specificity of 70.0% (95% CI, 34.8–93.3%) were observed at a threshold of 1:5 in serum. No false-positive cases were identified in patients with low CrAg titers in CSF and all positive predictive values (PPVs) were 100%. Among the cases with low serum CrAg titers, lumbar puncture was performed in 97 patients and positive CSF CrAg titers were reported in 6 patients. In conclusion, the results of this study imply that low CrAg LFA titer, either in serum or CSF, is crucial for early diagnosis of cryptococcosis in HIV-negative patients, and lumbar puncture is recommended to be performed routinely for CSF testing when a positive low serum titer is reported. Cryptococcal meningitis should be considered seriously when the CSF CrAg titer is positive.

## INTRODUCTION

Cryptococcosis is a life-threatening mycosis that primarily occurs in individuals with significant immunologic impairment, including those who have HIV infection, solid organ transplantation, hematological malignancy, and other diseases that affect cellular immunity (Pappas 2013, La Hoz & Pappas 2013). Moreover, cryptococcosis also occurs in individuals with no clinically recognized immunocompromising conditions (Zhu et al. 2010), with up to 20% phenotypically “normal” hosts in some clinical centers (Pappas et al. 2001), which contributes to a large worldwide disease burden. Consequently, early diagnosis of cryptococcosis is important, and a rapid yet reliable diagnostic test is needed.

The cryptococcal antigen (CrAg) lateral flow assay (LFA) is a recently developed dipstick sandwich immunochromatographic assay that uses gold-conjugated, monoclonal antibodies (mAbs) impregnated onto a test strip, which are highly reactive with CrAg across the range of all four cryptococcal serotypes (McMullan et al. 2012, Gates-Hollingsworth & Kozel 2013). Due to the use of mAbs, CrAg LFA is highly sensitive and specific for the detection of cryptococcosis in HIV-positive patients. In a large validation study in Africa, the CrAg LFA was performed in 832 HIV-positive patients, and the assay sensitivity of 99.3% and specificity of 99.1% were reported using cerebrospinal fluid (CSF) (Boulware et al. 2014). A satisfactory median sensitivity and specificity of 100% (95% CI, 95.6–100%) and 99.5% (95% CI, 95.7–100%), respectively, were also reported for serum specimens from HIV-infected individuals in a review including seven abstracts and two full-length articles (Vijayan et al. 2013). Nevertheless, as most comparative investigations have been conducted primarily using samples from HIV-positive patients, little is known about the relative diagnostic accuracy in HIV-negative individuals.

As an early diagnostic approach for cryptococcosis, higher serum CrAg titers were associated with an increased risk of concurrent cryptococcal meningitis in HIV-positive patients (Wake et al. 2018), and CSF CrAg titers ≥1:1280 were associated with significantly higher mortality (Kabanda et al. 2014). However, there are limited studies for the diagnostic accuracy of low CrAg titer (≤1:10). Only 2 culture-confirmed cryptococcal meningitis cases with low CrAg titers of 1:10 were reported in HIV-infected patients (Kabanda et al. 2014). For patients without HIV infection, a retrospective study carried out by the Mayo Clinic reported that 13 of 18 cases with low CrAg LFA titers were found to be false-positive (Dubbels et al. 2017). However, another study reported that 9 of 19 HIV-negative cases with low CrAg titer were identified to have pulmonary cryptococcosis (Erin et al. 2019). The limited data available made it necessary to evaluate the diagnostic accuracy of low CrAg LFA titers in HIV-negative patients.

## METHODS

### Study design and population

This was a retrospective study conducted using patient samples from January 2013 to December 2017 obtained in the Huashan Hospital, a tertiary hospital in Shanghai, to evaluate the diagnostic value of low CrAg LFA titers (≤ 1:10). The study population included HIV-negative, adult patients (≥ 18 years old) with low CrAg titers in either serum and/or CSF samples before receiving any antifungal treatment. HIV-positive individuals were excluded from this study. At the time of CrAg testing, serum and CSF CrAg titers were determined using the LFA test (IMMY Inc., Norman, Oklahoma) according to the manufacturer’s instructions.

### Clinical data

The electronic medical record system was used to retrieve the following patient demographics and clinical variables: age, sex, immune status, co-morbidities, other microbiological laboratory data (e.g., culture, histopathological findings, and/or serological results), final diagnosis, and antifungal treatment.

### Definitions

The CrAg LFA titer ≤1:10 was defined as low CrAg titer (Day et al. 2013). Cryptococcal infections were defined as either “proven”, “probable”, “possible” or “non-cryptococcosis”, as described for other invasive fungal diseases, with some modifications in patients with low CrAg LFA titers (De Pauw et al. 2008; Additional file 1: Table S1).

### Statistical analysis

To evaluate the diagnostic accuracy of low CrAg LFA titers, we combined and analyzed the following subgroups together: proven with probable cryptococcosis versus non-cryptococcosis. Individuals with possible cryptococcosis were excluded from the diagnostic accuracy analysis due to the indefinite diagnosis. The accuracy estimates were calculated as sensitivity, specificity, positive predictive value (PPV), and negative predictive value (NPV) for serum and CSF at different threshold titers, respectively. In each titer, the numbers of true-positive and false-positive patients were categorized into 2 groups according to their immune status. Statistical analysis was performed with Stata software, version 11.2 (StataCorp, College Station, Texas).

## RESULTS

### Patient characteristics

**Fig. 1:**
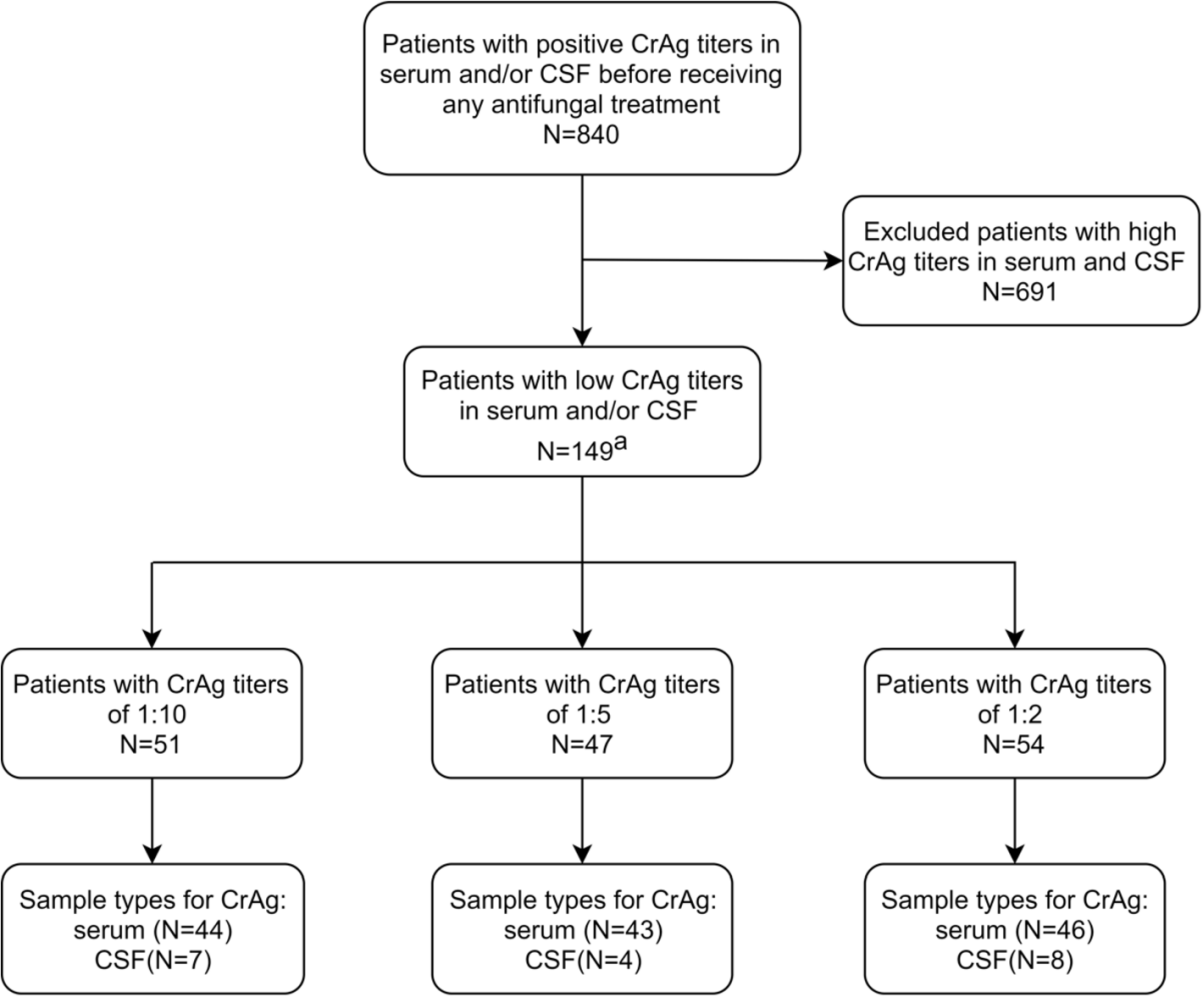
Flow-chart to show individuals included and excluded from the study. ^a^ Three of the 149 patients were tested of low CrAg LFA titers in both serum and CSF, resulting in 152 samples included. Abbreviations: CrAg, cryptococcal antigen; CSF, cerebrospinal fluid.

A total of 840 unique patients with 4496 positive specimens were screened, and 149 patients that had low CrAg titers (≤ 1:10) in serum and/or CSF were included in this study (Fig. 1). Of the patients enrolled, 94 were male and 55 were female, and the median age was 52 years (range, 20–88 years). One or more predisposing factors were found in 54 of the 149 patients (36.2%). The most common predisposing factors were solid organ tumors (*n* = 20) and steroid or immunosuppressants administration (n = 20); other predisposing factors and demographic characteristics are summarized in Table 1.

**Table 1:** Basic characteristics of patients with low CrAg LFA results

Variables	Patients with CrAg titers of 1:10 (*N* = 51)	Patients with CrAg titers of 1:5 (*N* = 47)	Patients with CrAg titers of 1:2 (*N* = 54)
Age, median year (range)	51 (21–74)	51 (24–84)	52.5 (20–88)
Sex, Female	13 (25.5)	22 (46.8)	22 (40.7)
Predisposing factors^a^	15 (29.4)	18 (38.3)	23 (42.6)
Autoimmune diseases	5 (9.8)	7 (14.9)	8 (14.8)
Corticosteroids or immunosuppressants administrations	7 (13.7)	5 (10.6)	10 (18.5)
Haematological malignancy	3 (5.9)	3 (6.4)	3 (5.6)
Lung cancer	2 (3.9)	2 (4.3)	3 (5.6)
Type 2 diabetes mellitus	0 (0.0)	1 (2.1)	3 (5.6)
Decompensated liver cirrhosis	0 (0.0)	1 (2.1)	1 (2.1)
Signs and symptoms			
Cough	21 (41.2)	16 (34.0)	17 (31.5)
Shortness	1 (2.0)	1 (2.1)	2 (3.7)
Haemoptysis	2 (3.9)	0 (0.0)	1 (1.9)
Chest pain	9 (17.6)	4 (8.5)	5 (9.3)
Fever	7 (13.7)	6 (12.8)	8 (14.8)
Headache or dizziness	6 (11.8)	3 (6.4)	6 (11.1)
Fatigue	1 (1.9)	5 (10.6)	2 (3.7)
Abdominal distention	0 (0.0)	1 (2.1)	0 (0.0)
Diagnosis^b^			
Proven	12 (23.5)	10 (21.3)	7 (13.0)
Probable	32 (62.7)	24 (51.1)	23 (42.6)
Possible	7 (13.7)	10 (21.3)	17 (31.5)
Non-cryptococcosis	0 (0.0)	3 (6.4)	7 (13.0)

Data are presented as No. (%) unless otherwise indicated

Abbreviations: *LFA* lateral flow assay; *CrAg* cryptococcal antigen; *CSF* cerebrospinal fluid; *NS* no significant

^a^Two of the 54 patients with predisposing factors were tested of low CrAg LFA titers in both serum and CSF

^b^Three patients were tested of low CrAg LFA titers in both serum and CSF. Patient 1 with a CrAg titer of 1:5 in serum and 1:10 in CSF was diagnosed as proven pulmonary cryptococcosis along with proven cryptococcal meningitis. Patient 2 with CrAg titer of 1:5 in serum and 1:10 in CSF was diagnosed as probable pulmonary cryptococcosis along with proven cryptococcal meningitis. Patient 3 with CrAg titer of 1:5 in serum and 1:2 in CSF was diagnosed as possible cryptococcal infection in both lung and CNS

Most patients (75.2%, 112/149) had a paired CSF sample collection near the time of serum drawn. Of those, 91 (81.3%) had a low serum titer and a negative CSF titer, 12 (10.7%) had a high serum titer (> 1:10) and a low CSF titer, 3 (2.7%) had a low serum titer and a high CSF titer, 3 (2.7%) had both low serum and CSF titers, and 3 (2.7%) had a low titer in CSF and a negative in serum. For the remaining 37 patients, most of them (*n* = 36) had only the serum CrAg LFA test alone; the remaining 1 patient had the LFA titer performed only in CSF.

### Diagnoses of Cryptococcosis

Patients with low CrAg LFA titers were categorized as “proven”, “probable”, “possible”, or “non-cryptococcosis” infections, according to the revised diagnostic criteria.

Proven pulmonary cryptococcosis was diagnosed in 24 patients, on the basis of positive respiratory tract cultures (*n* = 2), compatible histopathological findings (*n* = 21), or both (*n* = 1). The most common results in serum were titers of 1:5 (*n* = 10), followed by 1:10 (*n* = 8) and 1:2 (*n* = 6). Among the 5 patients with confirmed meningitis based on the isolation of *Cryptococcus* in culture (*n* = 5), 4 had CrAg titers of 1:10 in CSF, while 2 of them had CrAg titers of 1:5 in serum at the same time. The remaining 1 patient with proven cryptococcal meningitis had a CSF CrAg titer of 1:2. The median time to cultural negativity was 9 days (range, 2–15 days) and the median time to a declined CrAg titer was 10 days (range, 7–15 days) after timely antifungal treatment.

Probable pulmonary cryptococcosis were diagnosed in 30, 22, and 20 patients with titers in the serum of 1:10, 1:5, and 1:2, respectively. Probable cryptococcal meningitis was diagnosed in 3, 2, and 2 patients with titers of 1:2, 1:5, and 1:10, respectively. All serum and CSF low CrAg titers of proven and probable cryptococcosis are detailed in Table 2.

**Table 2:** Review of patients with proven and probable cryptococcosis

	Pulmonary Cryptococcosis		Cryptococcal Meningitis	
	Proven	Probable	Proven^a^	Probable
1:10	8 (33.3%)	30 (41.7%)	4 (80.0%)	2 (28.6%)
1:5	10 (41.7%)	22 (30.6%)	0 (0.0%)	2 (28.6%)
1:2	6 (25.0%)	20 (27.8%)	1 (20.0%)	3 (42.9%)
Total	24	72	5	7

Data are presented as No. (%) unless otherwise indicated

^a^Two proven cryptococcal meningitis patients with CSF titer of 1:10 and serum titer of 1:5 were concurrently diagnosed as proven and probable pulmonary cryptococcosis, respectively

Possible cryptococcosis were diagnosed in 33 patients, of whom 19 cases did not receive any antifungal regimen, and no laboratory or clinical evidence of cryptococcosis was found at the time of evaluation. Fourteen patients were lost to follow up.

**Fig. 2:**
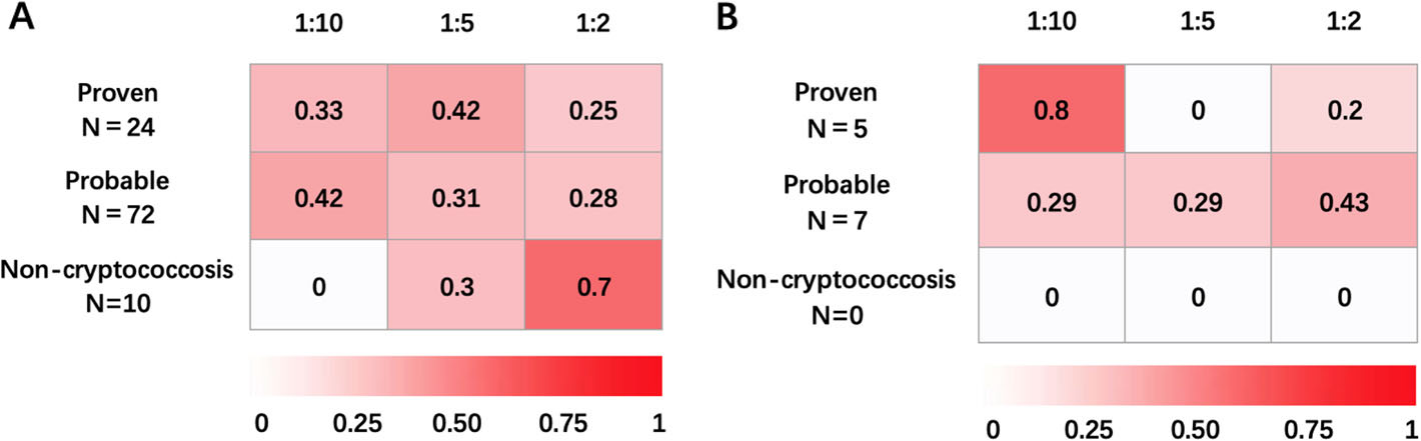
Heat map of patients with cryptococcosis and CrAg titers. **A***.* Heat map of patients with cryptococcosis and CrAg titers in serum. **B**. Heat map of patients with cryptococcosis and CrAg titers in CSF. The proportion of patients with cryptococcosis in each diagnostic classification and the corresponding CrAg titer value in serum or CSF are illustrated. The intensity of the red color increases with of greater proportion of patients falling with corresponding CrAg titer and diagnostic classifications. Abbreviations: CrAg, cryptococcal antigen; CSF, cerebrospinal fluid.

For the remaining 10 patients, cryptococcal infections were excluded and alternative diagnoses were ultimately established (Additional file 2: Table S2). Most of them had serum titers of 1:2 (*n* = 7), while 3 patients were reported to have serum titers of 1:5. The proportions of patients with low CrAg LFA titers for each diagnostic category are shown in Fig. 2. In serum, a titer of 1:5 is displayed as the highest intensity of red in proven cases, and 25% and 28% patients in proven and probable categories, respectively, were tested at 1:2. For CSF samples, the false positive category is colored with white in all titers, while medium red displays the probable diagnosis category with 43% of the patients at a titer of 1:2. One or more predisposing factors were found in 6 patients. Serum rheumatoid factor (RF) was positive in 2 cases, and invasive aspergillosis was identified in 3 cases (1 case of pulmonary aspergillosis and 2 cases of rhinosinusitis caused by *Aspergillus*). Overall, 10 patients were considered to have false-positive CrAg LFA results, all in serum, leading to an overall false-positive rate of 6.7% (10/149).

### Patients with elevated serial CrAg titers

Elevated serial CrAg titers were observed in 5 patients, of whom 1 was eventually diagnosed with proven pulmonary cryptococcosis, 1 with proven cryptococcal meningitis and 3 with probable pulmonary disease. Percutaneous lung puncture biopsy was performed in a patient with a serum titer of 1:2, for whom the histopathological and culture findings confirmed the diagnosis of pulmonary cryptococcosis, in which the CrAg titer increased to 1:640 after 84 days without antifungal therapy. For the female patients with proven cryptococcal meningitis, the initial CrAg titer was negative in serum and 1:2 in CSF, which deteriorated after 1 month to 1:160 and 1:2560 in serum and CSF, respectively. The dynamic changes in the serum titers of 3 probable pulmonary cryptococcosis patients were as follow: (1) from 1:2 to 1:10 before antifungal treatment, (2) from 1:10 to 1:20 before antifungal treatment and (3) from 1:10 to 1:80 after antifungal treatment.

### Diagnostic accuracy of low CrAg LFA titers

We showed the numbers of proven, probable and non-cryptococcosis patients grouped by different thresholds of low CrAg titers (Additional file 3: Table S3). Based on the grouping, the sensitivities and specificities of the low titers associated with a clinical diagnosis of cryptococcosis are summarized in Table 3. We first evaluated the performance characteristics of low CrAg titer at a threshold of 1:10 in serum. This showed a sensitivity of 39.6% (95% CI, 29.7–50.1%) and specificity of 100% (95% CI, 69.2–100%), and PPVs and NPVs of 100% (95% CI, 90.7–100%) and 14.7% (95% CI, 7.3–25.4%), respectively. At a threshold of 1:5 in serum, although the sensitivity of 72.9% (95% CI, 62.9–81.5%) was much higher, a declined specificity of 70.0% (95% CI, 34.8–93.3%) was observed. The PPVs in patients without predisposing factors were higher than those in patients with predisposing factors (98.1% versus 90.5%, respectively); no significant difference was found between the two groups.

**Table 3:** Diagnostic accuracy of different low CrAg LFA titers in patients with different immune status

Threshold	Value % (95% CI)			
	Sensitivity	Specificity^a^	PPV	NPV
Serum				
= 1:10	39.6 (29.7–50.1)	100.0 (69.2–100.0)	100.0 (90.7–100.0)	14.7 (7.3–25.4)
With predisposing factors	33.3 (16.5–54.0)	100.0 (54.1–100.0)	100.0 (66.4–100.0)	25.0 (9.8–46.7)
Without predisposing factors	42.0 (30.2–54.5)	100.0 (39.8–100.0)	100.0 (88.1–100.0)	9.1 (2.5–21.7)
≥ 1:5	72.9 (62.9–81.5)	70.0 (34.8–93.3)	95.9 (88.5–99.1)	21.2 (9.0–38.9)
With predisposing factors	70.4 (49.8–86.2)	66.7 (22.2–95.7)	90.5 (69.6–98.8)	33.3 (9.9–65.1)
Without predisposing factors	73.9 (61.9–83.7)	75.0 (19.4–99.4)	98.1 (89.7–100.0)	14.3 (3.0–36.3)
CSF				
= 1:10	50.0 (21.1–78.9)	/	100.0 (54.1–100.0)	0.0 (0.0–45.9)
With predisposing factors	33.3 (4.3–77.7)	/	100.0 (15.8–100.0)	0.0 (0.0–60.2)
Without predisposing factors	66.7 (22.3–95.7)	/	100.0 (39.8–100.0)	0.0 (0.0–84.2)
≥ 1:5	66.7 (34.9–90.1)	/	100.0 (63.1–100.0)	0.0 (0.0–60.2)
With predisposing factors	50.0 (11.8–88.2)	/	100.0 (29.2–100.0)	0.0 (0.0–70.8)
Without predisposing factors	83.3 (35.9–99.6)	/	100.0 (47.8–100.0)	0.0 (0.0–97.5)

Data are presented as % (95% confidence interval)

Abbreviations: *CI* confidence interval; *NPV* negative predictive value; *PPV* positive predictive value; *LFA* lateral flow assay; *CrAg* cryptococcal antigen; *CSF* cerebrospinal fluid

^a^Specificity was not calculated because no false positive case was found in patients with low CrAg LFA titer in CSF

Among the cases with low CrAg titers in CSF, all 12 subjects were diagnosed with proven or probable cryptococcal meningitis, yielding sensitivities of 50.0% (95% CI, 21.1–78.9%) and 66.7% (95% CI, 34.9–90.1%) at thresholds of 1:10 and 1:5, respectively. Of note, the PPVs were 100%, and no false-positive case was identified in patients with low CrAg titers in CSF.

## DISCUSSION

The aim of this study was to retrospectively evaluate the diagnostic accuracy of low CrAg titers in HIV-negative patients. Using patient samples that were positive for CrAg by the LFA methodology, we found that low CrAg LFA titers were commonly detected, with a prevalence of 17.7% (149/840) in our study. Similar to 32.3% (10/31) HIV-negative patients with confirmed cryptococcosis showing low titers in a previous study carried out by the National Institutes of Health (Jitmuang et al. 2016), low CrAg titers were also identified in 20.0% (5/25) proven or probable cryptococcosis patients through an investigation at the Mayo Clinic (Dubbels et al. 2017), indicating a potentially high incidence of low CrAg titer in HIV-negative patients, in whom the fungal burden may be lower.

Early diagnosis, usually confirmed by CrAg titers in serum or CSF, is crucial for reducing the dissemination, disability and mortality of cryptococcosis (Sungkanuparph et al. 2017). However, for individuals with low CrAg titers, the presenting symptoms are usually nonspecific, and antifungal treatment is often deferred until the disease has deteriorated (Wake et al. 2018, Aye et al. 2016). In our study, we evaluated the sensitivity of low CrAg LFA titers for the first time. Furthermore, in the heat map for the serum, all CrAg titers showed as red, and nearly a uniform color was observed in proven or probable diagnostic categories. Our results imply that all positive results, including titers of 1:2, deserve careful clinical consideration. Notably, elevated serum titers were identified in 5 proven or probable cases, and subsequent high CrAg titers (≥ 1:10) in serum were detected in all of them, suggesting that repeated dynamic tests and close follow-ups are necessary. In a large study including 19,233 individuals, Wake et al. reported that blood CrAg titer was significantly associated with concurrent cryptococcal meningitis in patients with or without headache (Wake et al. 2018). However, whether lumbar puncture should be performed routinely in patients with serum low CrAg titers remains controversial though asymptomatic cryptococcal meningitis was not uncommon (Liechty et al. 2007, Jarvis et al. 2009, Rajasingham et al. 2012, Vidal & Boulware 2015). Our study revealed that in 97 patients with low serum titers in whom lumbar puncture was performed, positive titers in CSF were reported in 6 patients. Among these 6 patients, low CSF titers were observed in 3 patients, including 2 proven and 1 possible cryptococcal meningitis cases. The remaining 3 individuals had CSF titers of > 1:10 and were all diagnosed with proven or probable cryptococcal meningitis. Moreover, compared with serum, the PPVs were 100% and no false positive case was identified in patients with low CrAg titers in CSF, which indicates a more powerful diagnostic value. Taken together, lumbar puncture should be performed routinely in all serum CrAg-positive individuals, given to the high PPV results in CSF titers.

Of note, our study showed that all false-positive cases were found in low serum titer cases, and more than a half (60.0%, 6/10) of them had predisposing conditions. Likewise, in the investigation by Dubbels et al., one or more co-existing predisposing factors were identified in 4 of 11 false-positive individuals with an overall rate of 36.4% (Dubbels et al. 2017). However, the role of the immune status in the diagnostic accuracy of the CrAg LFA test remains unknown, though the incidence of false positives was higher in patients with predisposing factors. In our study, a higher PPV in patients without predisposing factors at the threshold of 1:5 in serum was observed, but without a statistically significant difference, possibly because of the small sample size. Apart from the immune status, the other factors affecting false positives in the LFA are still unclear. Previous studies have reported that false positive results by the LFA may occur in patients with *Trichosporon asahii, Paracoccidioides brasiliensis,* and *Aspergillus* species infections (Vijayan et al. 2013, Rivet Dañon et al. 2015, Jitmuang et al. 2016, Dubbels et al. 2017). As for RF, the diagnostic interference of RF with the LFA has not been well recognized yet. We identified elevated RF in 2 patients with false positive results, which may be a contributing factor for false positives. Additionally, a 1:2 false positive result was found in a patient with African trypanosomiasis; cross-reaction with parasites in the CrAg LFA has not been reported before and it requires further investigation.

## CONCLUSION

Our study suggests that a low CrAg LFA titer is of significant value for early diagnosis of cryptococcosis. For low titers in serum, all positive results were valuable and suggest that repeated testing as well as close follow-ups are required. Lumbar puncture was also recommended to be performed routinely in patients with low serum CrAg titers, given to the high PPVs in CSF titers. As our study was limited by the retrospective design, insufficient sample size, and precluding patients with higher titers, an investigation with a larger sample size is needed to corroborate our findings.

## Supplementary information

**Additional file 1 Table S1.** Modified definitions of cryptococcosis among non-HIV patients with low CrAg LFA titers in serum and/or CSF.

**Additional file 2 Table S2.** Summary of patients with false-positive serum CrAg LFA results.

**Additional file 3 Table S3.** Numbers of patients grouped by different thresholds of low CrAg LFA titers.

## Data Availability

All data generated or analyzed during this study are included in this published article.

## Abbreviations

BALF: Bronchoalveolar lavage fluid
CrAg: Cryptococcal antigen
CSF: Cerebrospinal fluid
LFA: Lateral flow assay;
mAbs: Monoclonal antibodies
MRI: Magnetic Resonance Imaging
NPV: Negative predictive value
PPV: Positive predictive value
RF: Rheumatoid factors
