# Supplementary material for: Evaluation of low cryptococcal antigen titer as determined by the lateral flow assay in serum and cerebrospinal fluid among HIV-negative patients: a retrospective diagnostic accuracy study
Source: IMA Fungus. 2020 Mar 10;11:6. doi: 10.1186/s43008-020-00028-w (PMC7325107; doi:10.1186/s43008-020-00028-w)
Supplement: Supplementary file 1 — Additional file 1 Table S1. Modified definitions of cryptococcosis among non-HIV patients with low CrAg LFA titers in serum and/or CSF. [file 43008_2020_28_MOESM1_ESM.docx]

Additional file 1: **Table S1.** Modified definitions of cryptococcosis among non-HIV patients with low CrAg LFA titers in serum and/or CSF

| Pulmonary Cryptococcosis | | Cryptococcal Meningitis | | Possible Cryptococcosis | Non-cryptococcosis |
| --- | --- | --- | --- | --- | --- |
| Proven | Probable | Proven | Probable |  |  |
| 1. Positive culture or compatible histopathological findings of *Cryptococcus* from lung tissue   AND/OR:   1. Positive direct microscopy or culture of *Cryptococcus* from sputum or BALF | 1. Presence of respiratory symptoms or not   AND:   1. Pulmonary lesions on CT scan   AND:   1. Radiological improvement or decreased serous CrAg results after antifungal treatment   OR:  CrAg titers in serum rising to >1:10 with or without antifungal treatment | 1. Positive smear or culture findings of *Cryptococcus* from CSF   AND/OR:   1. Compatible histopathological findings of *Cryptococcus* in brain tissue | 1. Co-existence with CNS symptoms or not   AND:   1. Abnormalities in CSF cytology and biochemistry   OR:  high CSF opening pressure  OR:  Abnormal cranial imaging including MRI  AND:   1. Improved CSF conditions and/or radiological findings and/or LFA results after antifungal treatment   OR:  CrAg titers in CSF rising to >1:10 with or without antifungal treatment | 1. Unimproved Conditions without any antifungal treatment   OR:   1. Uncertain diagnoses due to loss of follow-up | 1. Improved conditions without any antifungal treatment including a declined or negative CrAg LFA result, and/or improved signs, symptoms and/or radiological findings   AND:   1. A definitive alternative diagnosis based on etiological, pathological or molecular biological examinations   AND:  No additional evidence of cryptococcal infections |

Abbreviations: LFA, lateral flow assay; CrAg, cryptococcal antigen; CSF, cerebrospinal fluid; BALF, bronchoalveoar lavage fluid; CNS, central nervous system; CT, computed tomography; MRI, magnetic resonance imaging.
