# Supplementary material for: Evaluation of low cryptococcal antigen titer as determined by the lateral flow assay in serum and cerebrospinal fluid among HIV-negative patients: a retrospective diagnostic accuracy study
Source: IMA Fungus. 2020 Mar 10;11:6. doi: 10.1186/s43008-020-00028-w (PMC7325107; doi:10.1186/s43008-020-00028-w)
Supplement: Supplementary file 2 — Additional file 2 Table S2. Summary of patients with false-positive serum CrAg LFA results. [file 43008_2020_28_MOESM2_ESM.docx]

Additional file 2**: Table S2.** Summary of patients with false-positive serum CrAg LFA results

| **No.** | **Predisposing factors^a^** | **CrAg LFA Titers** | | **RF^b^ (IU/ml)** | **Final diagnoses** | **Treatments** | **Follow-ups** |
| --- | --- | --- | --- | --- | --- | --- | --- |
|  |  | **serum** | **CSF^b^** |  |  |  |  |
| 1 | Rheumatoid arthritis, ISA agent use | 1:5 | ND | 18.2 | Rheumatoid arthritis | No antimicrobials treatment | LFA titer dropped to 1:2 in serum after 10 days |
| 2 | Post operation of lung cancer | 1:5 | Neg. | Neg. | Pulmonary aspergillosis | Voriconazole | No follow-up of LFA |
| 3 | / | 1:5 | ND | ND | Pulmonary tuberculosis | Isoniazid, rifampicin, pyrazinamide, ethambutol | LFA titer remained to be 1:5, chest CT scan improved after 4 weeks. |
| 4 | / | 1:2 | Neg. | Neg. | Invasive fungal rhinosinusitis (*Aspergillus*) | Itraconazole | Normal chest CT scan, symptoms improved after 3 days, LFA titer in serum turned to be negative after 5 weeks |
| 5 | Decompensated liver cirrhosis, CD4+ T lymphocyte proliferation, post splenectomy, | 1:2 | Neg. | Neg. | Brain abscess | Carbapenems | No follow-up of LFA, decrescent brain abscess |
| 6 | SLE, lupus nephritis, GCS and ISA agent use | 1:2 | ND | ND | SLE, lupus nephritis | No antimicrobials treatment | LFA titer in serum turned to be negative after 5 weeks |
| 7 | / | 1:2 | Neg. | Neg. | African trypanosomiasis | Eflornithine, nifurtimox | No follow-up of LFA, symptoms improved after 2 days |
| 8 | / | 1:2 | Neg. | Neg. | Pneumonia | Levofloxacin, moxifloxacin, cefuroxime, cefaclor | LFA titer remained to be 1:2 after 8 weeks, chest CT scan improved after 9 weeks |
| 9 | DM | 1:2 | Neg. | Neg. | Invasive fungal Rhinosinusitis (*Aspergillus*) | Voriconazole | LFA titer remained to be 1:2 after 7 weeks |
| 10 | ANCA-associated vasculitis and nephritis, GCS and ISA agent use | 1:2 | Neg. | 71.9 | ANCA-associated vasculitis and nephritis | No antimicrobials treatment | LFA in serum turned to be negative after 10 weeks, chest CT scan improved after 2.5 months |

Abbreviations: LFA, lateral flow assay; CrAg, cryptococcal antigen; CSF, cerebrospinal fluid; CT, computed tomography; RF, rheumatoid factors; SLE, systematic lupus erythematosus; GCS, corticosteroids; ISA, immunosuppressants; DM, diabetes mellitus; ANCA, Antineutrophil cytoplasmic antibody, HBV: hepatitis B virus; ND: not done; Neg. negative; No: Number.

^a^ Predisposing factor was not identified in No 3., No 4., No7., No. 8 patients.

^b^ Reference range: < 15.00IU/ml
