# Supplementary material for: Evaluation of low cryptococcal antigen titer as determined by the lateral flow assay in serum and cerebrospinal fluid among HIV-negative patients: a retrospective diagnostic accuracy study
Source: IMA Fungus. 2020 Mar 10;11:6. doi: 10.1186/s43008-020-00028-w (PMC7325107; doi:10.1186/s43008-020-00028-w)
Supplement: Supplementary file 3 — Additional file 3 Table S3. Numbers of patients grouped by different thresholds of low CrAg LFA titers. [file 43008_2020_28_MOESM3_ESM.docx]

Additional file 3: **Table 3.** Number of patients grouped by different thresholds of low CrAg LFA titers.

| **Threshold** | **Proven & Probable** | | | **Non-cryptococcosis** | | |
| --- | --- | --- | --- | --- | --- | --- |
|  | **Serum** | **CSF** | **Total** | **Serum** | **CSF** | **Total** |
| 1:10 |  |  |  |  |  |  |
| Titers of = 1:10 | 38 | 6 | 46 | 0 | 0 | 0 |
| With predisposing factors | 9 | 2 | 13 | 0 | 0 | 0 |
| Without predisposing factors | 29 | 4 | 33 | 0 | 0 | 0 |
| Titers of <1:10 | 58 | 6 | 65 | 10 | 0 | 10 |
| With predisposing factors | 18 | 4 | 23 | 6 | 0 | 6 |
| Without predisposing factors | 40 | 2 | 42 | 4 | 0 | 4 |
| 1:5 |  |  |  |  |  |  |
| Titers of ≥ 1:5 | 70 | 8 | 81 | 3 | 0 | 2 |
| With predisposing factors | 19 | 3 | 25 | 2 | 0 | 2 |
| Without predisposing factors | 51 | 5 | 56 | 1 | 0 | 1 |
| Titers of < 1:5 in serum | 26 | 4 | 30 | 7 | 0 | 7 |
| With predisposing factors | 8 | 3 | 11 | 4 | 0 | 4 |
| Without predisposing factors | 18 | 1 | 19 | 3 | 0 | 3 |

Abbreviations: CrAg, cryptococcal antigen; LFA, lateral flow assay; CSF, cerebrospinal fluid.
